# A Comparative Study of Data-Driven Models for Travel Destination Characterization

**DOI:** 10.3389/fdata.2022.829939

**Published:** 2022-04-07

**Authors:** Linus W. Dietz, Mete Sertkan, Saadi Myftija, Sameera Thimbiri Palage, Julia Neidhardt, Wolfgang Wörndl

**Affiliations:** ^1^Department of Informatics, Technical University of Munich, Garching, Germany; ^2^Research Unit E-Commerce, TU Wien, Vienna, Austria

**Keywords:** destination characterization, rank agreement metrics, expert evaluation, data mining, recommender systems, content-based filtering

## Abstract

Characterizing items for content-based recommender systems is a challenging task in complex domains such as travel and tourism. In the case of destination recommendation, no feature set can be readily used as a similarity ground truth, which makes it hard to evaluate the quality of destination characterization approaches. Furthermore, the process should scale well for many items, be cost-efficient, and most importantly correct. To evaluate which data sources are most suitable, we investigate 18 characterization methods that fall into three categories: venue data, textual data, and factual data. We make these data models comparable using rank agreement metrics and reveal which data sources capture similar underlying concepts. To support choosing more suitable data models, we capture a desired concept using an expert survey and evaluate our characterization methods toward it. We find that the textual models to characterize cities perform best overall, with data models based on factual and venue data being less competitive. However, we show that data models with explicit features can be optimized by learning weights for their features.

## 1. Introduction

The performance of data-driven systems is inherently determined by the underlying quality of data, which is becoming increasingly hard to judge in the current era of big data. When deciding on which features to use in the data model of an information retrieval or content-based recommender system, there are often several options to choose from. Out of the many options how to model a domain, how can one determine which instantiation of the available data is the *best?* The data-driven characterization of real-world items should capture each entity as closely as possible with respect to the user task supported by the system. As a principle, similar items in the physical world should also be similar in the information space, despite the loss of information and granularity. Thus, authors of content-based filtering algorithms (Pazzani and Billsus, 2007) should evaluate whether their data model matches the user goals, since a divergence might cause confusion and inevitably decrease the trust in and satisfaction with the system. Sometimes, the mapping to the physical world is obvious. When recommending a computer configuration, the feature values, such as the available memory or number of USB outlets have clear meaning and can be easily interpreted by the users and algorithms (Zhang and Pu, 2006). In other domains, however, a ground truth for items similarity is hard to capture, which is a fundamental problem (Yao and Harper, 2018): What are the movies most similar to “Fight Club”? Which cities are most similar to Munich? We as humans might have an intuition about such similarity concepts, but it is hard to develop recommendation algorithms that emulate such, possibly latent, concepts. Evaluating this is an under-researched challenge in the area of content-based recommendation, especially in the travel and tourism domain, where items such as destinations or travel packs are often not as clearly defined as consumer products. Approaches that rely on a history of explicit or implicit user interactions, such as collaborative filtering or bandit algorithms do not face this problem since there is a clear connection between the item and the user's rating (Su and Khoshgoftaar, 2009). Still, in cold-start situations, i.e., when too little interaction data is available for employing such approaches, a common strategy used is hybridization, which again requires using content-based algorithms to compute the initial recommendations (Çano and Morisio, 2017).

To make these considerations concrete, take a destination recommender system as an example. The CityRec system (Dietz et al., 2022), allows users to refine their travel preferences based on six features that were obtained and derived from various sources such as Foursquare and open data portals: “Nightlife,” “Food,” “Arts and Entertainment,” “Outdoor and Recreation,” “Average Temperature,” and “Cost.” However, when developing this system, we faced the issue of determining which data set and features are the most accurate and useful for prospective travelers to reason about destinations. Given that recommendations are computed using the cities' features and the users base their decisions on them, inaccuracies in the data model negatively impact the trust in the system. To the best of our knowledge, this two-fold challenge of choosing accurate data sources to quantify specific aspects travel destinations, as well as choosing which features to incorporate in a content-based recommender system has not been analyzed in a systematic way (Yao and Harper, 2018). This motivated us to develop this toolbox of methods to compare data sources with each other and also with respect to what is important in the domain of such a recommender system.

To make our contributions generalizable for different data models in various domains, we rely on rank agreement methods (Kendall, 1970), which operate on ranked lists based on the similarity measure of the recommender system. The proposed methods quantify correlations between conceptually diverse characterization methods to enable informed decision making with respect to which one to employ. For example, if it turns out that two characterization methods are highly correlated, i.e., both capture the same underlying concepts using different features, one could go ahead and exchange one for another without introducing disruptive changes in the resulting recommendations.

Furthermore, we propose a method to assess the quality of data models with respect to a *desired* concept. We argue that a destination recommender system should use a data model that results in recommendations that emulate the destination experience as closely as possible. To achieve this, it is imperative to assess which available data source and feature set approximate the concept of touristic experience best. However, such a “*gold standard”* is readily not available and typically can only be elicited for a small subset of the recommendation items. We elicit the concept of touristic experience using an expert study and propose methods to assess the quality of the characterization methods with such incomplete information.

To showcase the utility of our approach, we exercise the methods within the particularly challenging domain of content-based destination recommendation (Le Falher et al., 2015; Liu et al., 2018), where recommendations are solely computed based on the items' characteristics as opposed to rating or interaction data in collaborative filtering approaches. For this, we introduce 18 destination characterization methods for 140 cities, which we have collected from literature or constructed ourselves. Using well-established rank correlation methods (Kendall, 1970), we compute their pairwise similarities, thereby revealing families of similar data sources. To evaluate the data sources with respect to how tourists experience a destination, we conduct an expert study to elicit this latent concept. Using variants of established top-*k* rank agreement methods, we are able to assess the quality of the data sources by their similarity to the expert opinions.

The main contributions of this work are as follows:

- We propose a method to assess the similarities and the quality of data models characterizing items in content-based recommender systems.
- We introduce, instantiate, and compare 18 different destination characterization methods using the proposed methodology.
- We conduct a survey among travel experts to establish a similarity baseline of the different destination characterization methods. Using this expert-elicited concept, we assess and optimize the data sources with respect to this concept.

While we use destination characterization as our running example, our methods are not specific to this domain, since the proposed methods operate on ranked lists of any kind.

The structure of the paper is the following: after discussing the prior work in Section 2, we provide a description of the data for destination characterization. In Section 4, we introduce our methodology of how we made the data sources comparable using rank agreement metrics. The expert study in Section 5 shows how we elicited our desired concept. In Section 6, we present the analysis of which data sources capture similar concepts and which approximates the concept of touristic experience best. Finally, we conclude our findings and point out future work in Section 7.

## 2. Related Work

In recommender systems research, most algorithms traditionally use the collaborative filtering paradigm, i.e., interpreting user ratings (or similar explicit feedback) of items. However, in cold-start recommendation situations, where such interaction data does not exist to sufficient degree, content-based algorithms (Lops et al., 2011) play a role to be able to generate meaningful and personalized recommendations to the users. This research is motivated by practical challenges of content-based information retrieval systems, especially personalized recommender systems.

Concerning the similar item recommendation problem, Yao and Harper have shown that content-based algorithms have outperformed ratings- and clickstream-based ones with respect to the perceived similarity of items and the overall quality of the recommendations in the movie domain (Yao and Harper, 2018). Our work goes one step further: We provide a framework that allows to compare different data models of the same items with respect to the similarity according to a desired concept and additionally provide means to optimize the feature weights to approximate the concept even better.

We use the travel and tourism recommendation domain as a running example; however, other domains face similar challenges. In their survey, Borràs et al. (2014) identify four different tasks that tourism recommender systems have to cope with: recommending travel destinations or travel packages (Liu et al., 2011), suggesting attractions (Massimo and Ricci, 2018; Sánchez and Bellogín, 2022), planning trips (Gavalas et al., 2014; Dietz and Weimert, 2018), and accounting for social aspects (Gretzel, 2011). We aim to contribute to the feature engineering challenges of the first task: recommending travel destinations. We focus on the characterization of destinations, which is the task to establish the underlying data model for destination recommender systems. Herein, “destination” refers to cities. Key challenges in recommending cities are the intangibility of the recommended item, high consumption costs, and high emotional involvement (Werthner and Ricci, 2004).

Burke and Ramezani (2011) suggest the content-based recommendation paradigm as one of the appropriate ones for the tourism domain. Content-based recommenders need a domain model and an appropriate distance measure to enable effective matchmaking between user preferences and items to generate recommendations without details rating or interaction data. For this reason, they can be successful in situations where the interaction with the recommender is very rare and short-term and the user model can be derived from alternative information sources. Domain models in recommenders have been constructed using various data sets (Dietz et al., 2019) derived through analyses and user studies (Neidhardt et al., 2014, 2015) or realized through ontologies (Moreno et al., 2013; Grün et al., 2017). In this work, we compare different data-driven destination characterization methods, which project destinations onto the respective search spaces using different types of data, cf. Section 3.

Naturally, the question of which features are useful to characterize a destination for efficient retrieval in an information system arises. Dietz (2018) mentions challenges of characterizing destinations: the destination boundaries must be clearly defined, the data needs to be kept up-to date, and the features should be relevant with respect to the recommendation goal. Analyzing LBSN data to characterize cities and their districts has been an active topic in previous years (Silva et al., 2019). It has been shown that such data is quite useful to unveil characteristics of certain districts within a city (Le Falher et al., 2015). McKenzie and Adams (2017) suggested the use of Kernel density estimation models of check-ins to identify thematic areas within a city.

A related line of research is concerned with capturing and visualizing intangible concepts in urban areas: Quercia et al. (2015) used LBSN data and Google Street View imagery^1^ to determine intangible concepts such as the smell, the soundscape (Aiello et al., 2016), and general happiness (Quercia et al., 2014) on a street granularity. Analogously, street imagery can also be reliably used to measure distributions of income, education, unemployment, housing, living environment, health and crime as Suel et al. (2019) have demonstrated. Finally, it has been shown that it possible to automatically distinguish cities based on their architectural elements learned from street imagery (Doersch et al., 2015). Using features derived from such approaches could also be used to compute similarities of cities and their districts. Obtaining Google Street View images is feasible on a small and medium scale, however, the costs to do such an analysis on a global scale prevented us from experimenting with this data source.

In the area of content-based characterization of destinations for use in recommender systems there are so far few approaches. Sertkan et al. (2017) characterized a huge data set of 16,950 destinations based on 26 motivational ratings and 12 geographical attributes within the Seven Factor Model of tourism motifs. They proposed a cluster analysis and regression analysis to map the destinations to the vector space of the Seven Factor Model (Neidhardt et al., 2015). The framework was recently also used by Grossmann et al. (2019) to elicit preferences of prospective tourists using picture of destinations While modeling the user's interests using travel-related pictures has been shown to be possible, obtaining a representative set of images of global destinations in an automated fashion is an open research problem (Sertkan et al., 2020a,b). The development of the CityRec recommender system (Dietz et al., 2019, 2022) partly motivated investigating different data models for recommender system: CityRec uses a domain model based on Foursquare venue categories and further information such as a cost index or climate data collected from web APIs (Dietz et al., 2019). This data model is used both to elicit user preferences via conversational refinement, i.e., turn-based adjustment of the preferences in a dialogue with the system and to compute the recommendations in a content-based way.

It is striking that researchers invest a lot of energy into capturing signals from various online sources to approximate complex, intangible concepts. To the best of our knowledge, these data models are rarely systematically verified as to what extend they approximate the recommendation domain. In this paper, we propose a collection of several methods to provide researchers with tools to evaluate this.

## 3. Data Sources

To characterize destinations, we used various online data sources showcased in Table 1. Our selection criteria for the data sources were that they should have touristic relevancy, i.e., prospective travelers should be able to use them to familiarize themselves with a destination, or that they are already part of travel-related information systems, as is the case with the data set from Foursquare and the data sources in the factual category.

**Table 1:** Overview of the data sources for characterizing cities.

**Type**	**Name**	**Category**	**Data objects**	**Number of objects**	**Acronym**
Venue	Foursquare	LBSN	Venues	2,468,736	FSQ
Data	OpenStreetMap	Collaborative map	Map entities	3,106,856	OSM
Textual	Wikipedia	Collaborative encyclopedia	Documents	1,150,719 words	WP
	Wikitravel	Travel-related Wiki	Documents	984,777 words	WT
	Google travel	Travel information	Documents	56,499 words	GT
Factual	Webologen	Travel information provider	City features	49 tourism facts/city	TF
	Nomad list	Collaborative travel information	City features	8 features / city	Nomadlist
	Seven factor model	Scientific characterization	Derived factors	7 factors / city	7FM-2018
	Geographic location	Geographic location	Latitude, longitude	1 coordinate pair / city	GEO

**Figure 1:**
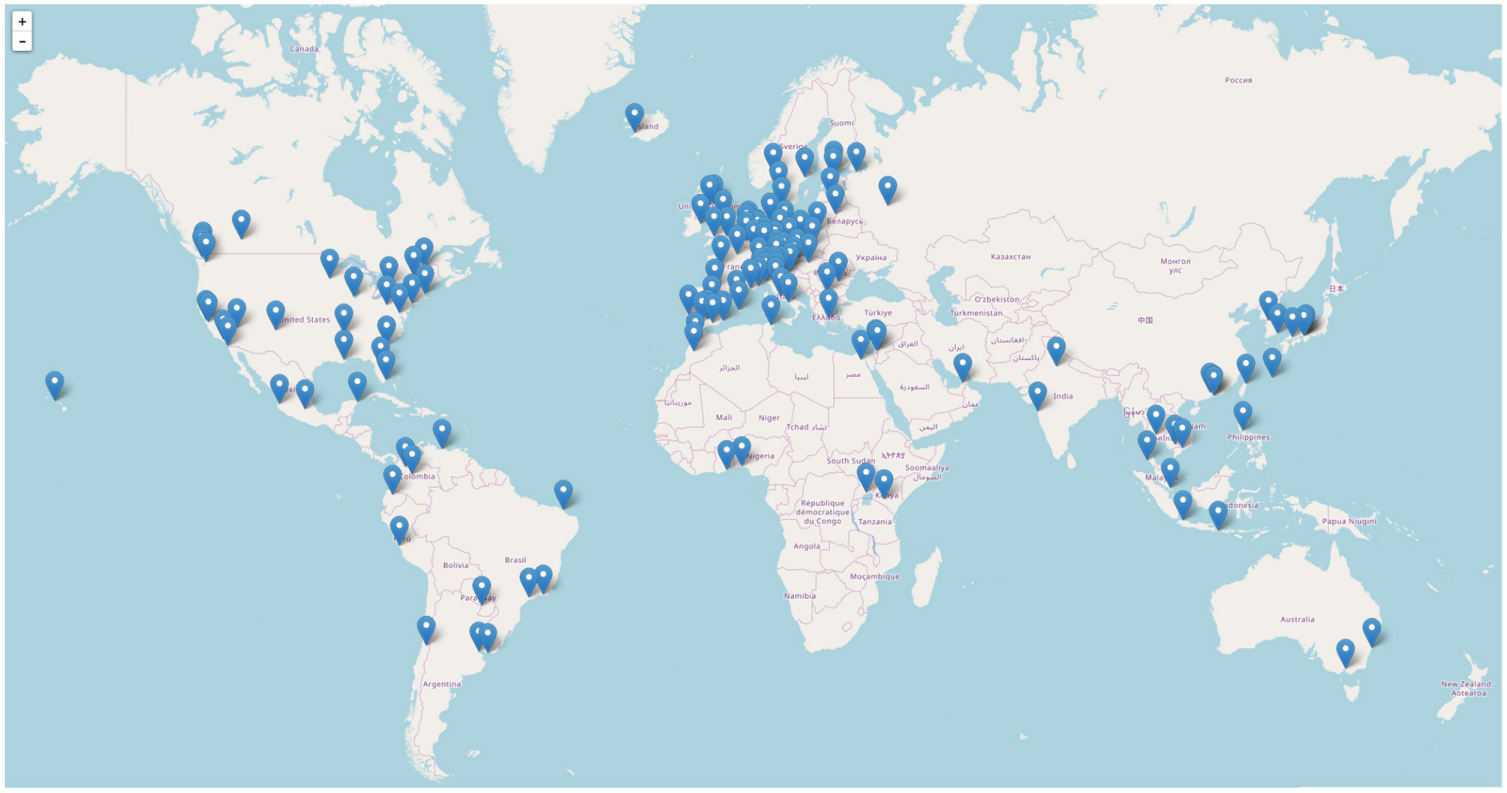
Geographic distribution of the characterized cities. Map data © OpenStreetMap contributors, see https://www.openstreetmap.org/copyright.

To obtain a balanced collection of destinations on all continents that would reflect the cultural differences of the travel destinations, we initially gathered an extensive list of prominent cities such as capital cities or relevant travel destinations. Unfortunately, not all cities could be characterized with all methods. Several destinations were not included in Nomad List and OpenStreetMap did often not have proper city boundaries for several cities in Asia and Africa. Our proposed approaches to make the destination characterization methods comparable, however, require complete data for each city. Thus, our data set for this study comprises a set of 140 cities, which are those that could be characterized with all data sources. Looking at Figure 1, the distribution of destinations on the planet is missing cities in Central Asia and Africa that had to be excluded due to this requirement, otherwise the distribution would roughly correspond to the world's population density. The full list is available in the replication pack.

Throughout this paper, we work with ranked lists of the cities most similar to one city. Such lists are based on one data source and start with the base city followed by the 139 other cities. This means that for each data source there are 140 such ranked lists. In the following, we describe the data sources and how we computed the similarity metrics between the cities.

### 3.1. Venue Data

The first type of data we used to characterize destinations is venue-based data. Intuitively, the variety of venues one can visit at a destination might reflect the experience of a traveler. The underlying assumption is that a destination can be characterized using the distribution of all its touristic venues. The following characterization methods rely on the assumption that the larger the variety of, e.g., restaurants or cultural sites of a city is, the better the score should be in these categories. This also means that we do not aim to assess the quality of the venues, since most venues do not come with quality indications such as ratings.

#### 3.1.1. Foursquare

Foursquare is a LBSN that offers a rich, well-structured taxonomy of venue categories and also allows reasonably generous API rate limits to crawl data from it. Using the “search venues” endpoint^2^, we were able to obtain a collection of each city's venues using a recursive algorithm that exhaustively queried all Foursquare venues specified within a bounding box. Using this method, we collected 2,468,736 venues in 140 cities that had at least 5,000 venues each.

**Figure 2:**
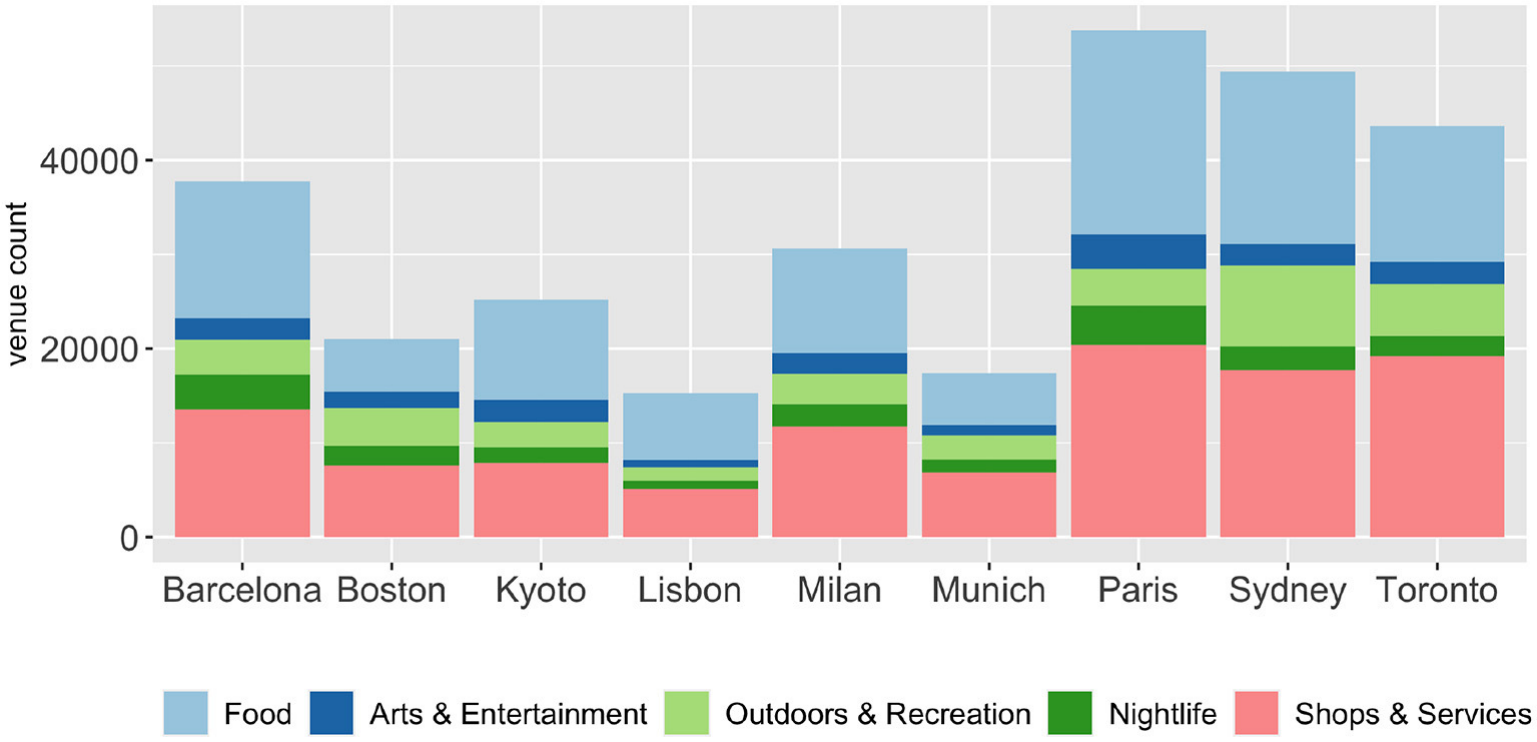
Venue category distribution for a subset of cities.

To create the specific set of lists, we needed to establish an association between the cities and the venue types. Foursquare provides a well-defined venue category hierarchy^3^, which allows us to map every venue to a top-level category, e.g., Science Museum → Museum → Arts & Entertainment. We use the tourism-related subset of these categories to create a feature set that enables us to characterize the cities, shown in Figure 2. These features can be conceptualized as a multi-dimensional vector space, however, to perform reasonable comparisons the data must be normalized to make large and small cities comparable. By normalizing the number of venues in each category using the total venue count of the city, we obtain the percentage of each category in the city's category distribution. This approach relies on the assumption that a larger number of venues in a certain category improves the touristic experience while visiting it. A simple example helps in demonstrating this: The cities in our data set have a certain distribution of venue categories; if the number of venues labeled with “Arts & Entertainment” in a city is on the high end of that distribution, it can be assumed that it likely offers a larger number of opportunities and should, thus, get a higher score in this category. Figure 2 shows the category distributions of a few cities of different continents and sizes that we have chosen as illustrative examples. Note that, unlike in the data model, this visualization is not normalized with respect to the number of venues. Examining Figure 2, one can see that many cities have a somewhat similar distribution of venue categories, where “Food” and “Shops & Service” dominate in general. To eliminate this effect, we apply min-max scaling to the calculated percentages. This way we obtain the final city scores for each of the features, which take values in [0, 1].

Using this method, we constructed two data models from Foursquare. The first on the four top-level categories – “Arts & Entertainment,” “Food,” “Outdoors & Recreation,” and “Nightlife” – and another one using the 337 second-level categories as aggregation target.

#### 3.1.2. OpenStreetMap

With OSM, we used a similar approach as with Foursquare. To obtain all map features, we set up our own OSM server and developed a querying client to obtain the entities within the city relations. The map entities are again hierarchically categorized on three levels^4^. The 27 top-level categories are subdivided into several subcategories which finally contain 1,032 types of map features. For example, the “amenity” category has several subcategories, such as “Healthcare,” “Transportation,” and “Entertainment, Arts and Culture.” These subcategories again contain numerous entities, uniquely identified by the full path, for example “amenity:entertainment/arts” and “culture:cinema.” As opposed to the Foursquare characterization, we also had exact city boundaries, so we could compute the area of the destination.

Leveraging this hierarchy, we again built a top-level model, which collapses the map entities to tourism-related categories: “tourism,” “leisure,” “historic,” “natural,” and “sport” as well as the venue count and the area. The second-level model comprises entities of 14 tourism-related subcategories as well as the venue count and area.

### 3.2. Textual Data

Using document similarity assessments such as the Jaccard Distance, Word2Vec embeddings and a transformers-based approach on texts describing a destination, we are able to compute pairwise similarities between the cities. As textual basis, we used three online resources: Wikipedia, Wikitravel, and Google Travel.

#### 3.2.1. Wikipedia

We used the articles of the English Wikipedia^5^ about the 140 cities to compute the similarity between cities. The mean length of the articles about our destinations was 8,219 words.

#### 3.2.2. Wikitravel

This collaborative travel guide^6^ provides useful information about touristic destinations. It is free of charge and offers detailed information about possible activities, recommended restaurants, and general advice for traveling. Some cities have sub-pages about their districts, however, we have only used the main articles to maintain comparability. The mean length of the articles was 7,034 words.

#### 3.2.3. Google Travel

Another popular platform for learning about travel destinations and planning trips is Google Travel^7^. Based on actual traveler visits and local insights, the platform provides a list of most iconic attractions. In this work, we used the short description of each attraction at a destination. For example, the description of *Schönbrunn Palace* in Vienna is “*Baroque palace with opulent interiors.”* Since there are often myriads of attractions in a city, we concatenated these descriptions into one document to obtain the overall description of the considered city. This resulted in one document per city with a mean length of 404 words.

#### 3.2.4. Text Processing and Similarity Measures

For the raw text of the three text sources, we used the same pre-processing steps. After the HTML tags were removed, the text was put in lower case and stripped of all special characters, such as line breaks and punctuation marks. Then, the terms were tokenized using a standard word tokenizer and the stop words eliminated to reduce noise.

For the Jaccard models, the document term matrix was computed based on the cleaned text and then the similarity between the cities was computed using the Jaccard Distance.

We use pre-trained Word2Vec and BERT-based models as zero-shot encoders to embed the documents. In case of the Word2Vec-based models, we aggregated the pre-trained word embeddings using mean-pooling to obtain the document embedding and used the cosine similarity to compute the similarity. We utilize the open-source library *spaCy*^8^ and in particular the english-core-web-large model, which outputs 300-dimensional vectors and is trained on *OntoNotes 5* (Weischedel et al., 2011), *ClearNLP Constituent-to-Dependency Conversion* (Choi et al., 2015), *WordNet 3.0* (Miller, 1995), and *GloVe Common Crawl* (Pennington et al., 2014). This means, that there was no need to fine-tuning the models; the default hyperparameters of Spacy could be re-used.

The BERT-based sentence encoder (Yang et al., 2021) we employed was also pre-trained on Wikipedia and Common Crawl^9^ to encode the documents, thus, again making further fine-tuning of (hyper-) parameters obsolete. We use the cosine similarity to rank the cities. Thus, we obtained nine textual ranked lists: three data sources × three similarity measures.

### 3.3. Factual Data

The third category is factual data with a focus on travel and tourism. This group comprises data sources that had readily available facts about destinations, such as rated features or geo-social features relevant for travelers. However, this does not imply that the quality of the data is beyond scrutiny.

#### 3.3.1. Webologen Tourism Facts

The former German eTourism start-up Webologen compiled a data set of 30,000 cities, which are described by 22 geographical attributes and 27 “motivational” ratings. The geographical attributes have binary values indicating the presence or absence of various geographical attributes: *sea, mountain, lake, island, etc*. The motivational ratings were assessed using proprietary methods at Webologen, taking into account infrastructure, climate, marketing, and economic data. With a score between 0 and 1, the motivational ratings, such as *nightlife, wellness, shopping, nature and landscape*, measure the quality of those touristic aspects at a destination. The higher the value, the better this aspect is for a traveler. Given this multitude of features, this data set provides a very detailed image of a tourism destination. Since there are multiple types of data (i.e., binary and interval scale), we use the Gower Distance (Gower, 1971) to compute the similarity for the city rankings.

#### 3.3.2. Nomad List

As opposed to Webologen's approach, Nomadlist^10^ employed a mixture of own data modeling and crowdsourcing to characterize cities for their suitability for digital nomadism. Built as a specialized platform for this community, it offers rich information about the cities in its database. We crawled the publicly available data and were able to obtain the following features for each city: “*Nomad Score,” cost, fun, life quality, air quality, healthcare, happiness, and nightlife*. Since these features were already available in a normalized interval format, we used the Euclidean Distance to compute the city similarity rankings.

#### 3.3.3. Seven Factor Model

This model was previously developed by Neidhardt et al. (2014, 2015) to capture the preferences and personality of tourists, but also to project touristic recommendation items such as destinations and attractions. Both user preferences and items are embedded into the same vector space using seven orthogonal dimensions: *Sun and Chill-Out, Knowledge and Travel, Independence and History, Culture and Indulgence, Social and Sports, Action and Fun, Nature and Recreation*. These factors were derived from a factor analysis of the well-known “Big Five” personality traits (Goldberg, 1990) and 17 tourists roles of traveler behavior (Gibson and Yiannakis, 2002). In subsequent work, they showed that tourism destinations can be mapped onto the Seven Factor Model using tourism facts based on the Webologen data set (Sertkan et al., 2019). We used the same mapping mechanism to reproduce the Seven Factor representations for each destination in our data set. Given that the resulting representation is a seven-dimensional vector [0, 1], we use the Euclidean Distance to compute the city similarity rankings.

#### 3.3.4. Geographical Distance

The geographic position of the destinations certainly also plays a role assessing the similarity among them. Intuitively, cities close to each other might have a higher similarity than those far apart. While this model might not provide much insight into the characterization of destinations, it still serves as an interesting baseline in assessing the similarities of other methods. We used the Haversine Distance (Robusto, 1957) based on the cities' geographic coordinates to compute this distance.

## 4. Comparing Ranked Lists

Each data source described in the previous section establishes a pairwise similarity for all cities. Selecting a city, we can rank all other cities based on these similarity scores. We want to compute metrics that capture the similarity of ranked lists, thus, revealing which data models capture a similar concept. In literature, one can find various methods to compute the agreement of two ranked lists. They are also known as rank “correlation” methods and essentially capture a notion of similarity between the ordering of items within two lists. For complete permutation groups, i.e., both lists have the same items and the same length, there are several established metrics, such as the Kendall's Tau Distance (Kendall, 1970), Spearman's Footrule Distance (Spearman, 1906), and Spearman's ρ (Spearman, 1904). Based on these measures, myriad other methods have been proposed to cater the needs of more specialized domains and other assumptions.

To precisely describe the methods, we briefly discuss our assumptions and introduce a terminology that is inspired by Fagin et al. (2003). Throughout this work, we consider ranked lists of 140 cities, which are our *fixed domain* *D*. We analyze several data models, which express their similarity in form of *ranked lists* rl∈RL, of which we ultimately would like to find which would be most suitable to be employed in a content-based recommender system. Each *ranked list* is a *permutation* of the set of permutations *S*_*D*_ of *D*. *rl*(*i*) denotes the rank of a city *i* in the ranked list *rl*. *rl*(1) is always the city based on which the model was created.

### 4.1. Rank Agreement of Complete Ranked Lists

The simplest problem to determine the correlation between two ranked lists is comparing two permutations (Kendall, 1970; Diaconis, 1988). We will briefly recapitulate two common measures for this, as they are the foundation of our proposed metrics for the agreement of top-*k* lists with a full permutation.

#### 4.1.1. Kendall's Tau Distance

It is defined as the minimum number of pairwise adjacent transpositions needed to transform one list into the other (Kendall, 1970). It counts the number of pairs of items *P*(*i, j*), such that *rl*_1_(*i*) < *rl*_1_(*j*) and *rl*_2_(*i*)>*rl*_2_(*j*). This is equivalent to the number of swaps required for sorting a list according to the other one using the Bubble Sort algorithm (Lesh and Mitzenmacher, 2006).


$$\begin{array}{l} T\left(rl_{1},rl_{2}\right)=\underset{i,j\in P}{\sum }{\bar{T}}_{i,j}\left(rl_{1},rl_{2}\right), \end{array}$$

where *P* = {{*i, j*}|*i*≠*j* and*i, j*∈*D*}, and ${\bar{T}}_{i,j}\left(rl_{1},rl_{2}\right)=1$ if *i* and *j* are in the opposite order, and 0 otherwise.

#### 4.1.2. Spearman's Footrule Distance

Intuitively, this metric is defined over the distance of the ranks of the same item in the two lists (Spearman, 1906).


$$\begin{array}{l} F\left(rl_{1},rl_{2}\right)=\overset{n}{\underset{i=1}{\sum }}|rl_{1}\left(i\right)-rl_{2}\left(i\right)| \end{array}$$

Despite being conceptually different it has been shown that in practice, both metrics yield similar results for full permutations (Kendall, 1970). In our evaluation in Section 6, we will use them to determine which data models capture similar underlying concepts and they form the foundation for our proposed rank agreement methods of incomplete rankings.

### 4.2. Rank Agreement of Incomplete Rankings

In their original definition, the rank agreement methods introduced in Section 4.1 are defined over two complete permutations of the same finite list. This assumption does not generally hold, since we were unable to characterize all cities with all data sources resulting in missing characterization of cities. We sidestepped this problem by considering only a subset of destinations that could be characterized with all methods.

#### 4.2.1. Problem Formulation

The outcome of the expert study (cf. Section 5) is a collection of top-*k* lists. Each top-*k* list contains the *k*≥10 most similar cities to the city the expert characterized. To find out which data model is the most similar to the experts' opinions, we need to modify the rank agreement methods to cope with this scenario. Concretely, this means that we need to compute the rank agreement between an expert's top-*k* ranking τ, where 10 ≤ *k* ≤ 139 and a complete permutation of length 140. To the best of our knowledge, we are first to systematically analyze this special case.

#### 4.2.2. Approaches in Literature

In literature, similar problems have been tackled in the area of biostatistics and information retrieval. Critchlow was first to establish a theoretical basis for such rankings (Critchlow, 1985), assuming a fixed domain of items *D*. One of the most comprehensive papers on the rank agreement of top-*k* lists is the one of Fagin et al. (2003). Unlike Critchlow and us, they did not assume a fixed domain of items and, thus, proposed very general distance measures for top-*k* lists that are not directly useful to our scenario. The authors also proved that in the general case, the measures for top-*k* lists reside in the same equivalence class and showcased further applications of these measures in the context of the rank aggregation problem (Dwork et al., 2001; Lin and Ding, 2008).

An important property of Kendall's Tau and Spearman's Footrule is that all ranks are treated equal, i.e., they do not take the potentially non-uniform relevancy of top-ranked or bottom ranked into account. In many domains, the assumption of uniform relevancy does not hold, thus, several other measures have been proposed. Iman and Conover (1987) proposed a concordance measure that prioritizes rank agreements at the top of the rankings, while Shieh proposed a weighted variant of Kendall's Tau, where the analyst can prioritize either low-ranked or high-ranked items (Shieh, 1998). The Average Precision (AP) correlation is another important measure in information retrieval that more heavily penalizes differences of top-ranked items compared to Kendall's Tau (Yilmaz et al., 2008).

In our domain at hand, the issues motivating the aforementioned papers are not present. Since our top-*k* lists are very short, we do not need to come up with additional weights based on the position within the list. Furthermore, given the underlying data sources and similarity measures used, the probability of tied ranks in the lists is very low so that this case can be neglected as well (Urbano and Marrero, 2017).

The alternative to dealing with the rank agreement problem of a top-*k* list and a permutation would be to disregard the inherent order of the ranked list and view it as a set. This would open the door to interpret each element of the list as an independent query, on which traditional information retrieval metrics can be computed such as Precision or the Reciprocal Rank. By repeating this process for each item, one could assess the quality of the expert's selection just as it is frequently done with search engines or recommender systems resulting in metrics, such as the Precision (Precision@K) or the Mean Reciprocal Rank (MRR). Instead, we aim to retain the ranking information by the experts and discuss various methods to compute metrics that operate on the ranked list semantics.

#### 4.2.3. Proposed Methods

Under the assumptions that 1) we have a fixed domain of items, and 2) only the relative ranking in the ranked lists matters, i.e., the concrete values of the agreement are not of importance, there is the option to randomly fill the missing items of a top-*k* list τ with the remaining items {*D*−τ} (Ekstrøm et al., 2018). This essentially constructs two permutations, which can then be assessed with the standard metrics from Section 4.1. By repeating this process a large number of times, the effect of the random items at the tail of the list is eliminated and, finally, the ranking is computed based on the mean value of all iterations. This simple idea would be a permissible option for our scenario; however, it requires much overhead computation and does not provide concrete values for the rank agreement, since only the top *k* items contribute to the signal, while the remaining ones are pure noise.

Thus, an analytic solution for this problem would be preferable. In our scenario, we can always assume that we have a fixed domain of items, since each data model will be able to produce similarity scores between all items. Thus, our problem is similar to the one Fagin et al. resolve in their approach, i.e., comparing one top-*k* list τ of length *k* with a ranked list *rl* (Fagin et al., 2003, Section 3.1), however, due to the fixed domain assumption, we only need to discriminate three cases. This results in a simpler problem without any room for uncertainty that might arise from having items that are in one top-*k* list, but not in the other.

- Case 1: *i*∈τ, and *rl*(*i*) ≤ *k* (the item is in the top-k list and the rank of the item in the permutation is at most *k*)
- Case 2: *i*∈τ, but *rl*(*i*)>*k* (the item is in the top-k list but the rank of the item in the permutation is greater than *k*)
- Case 3: *i*∉τ (the item is not in the top-*k* list)

Using this insight, we propose variants to Kendall's Tau and Spearman's Footrule distance for top-*k* lists.

- **Modified Spearman's Footrule Distance**
  If a city is in the top-*k* list (Case 1 & 2), we can compute the distance between τ(*i*) and *rl*(*i*) as before, since all information is still available. In Case 3, we do not add any penalty, since we have no information about which penalty should be applied. Thus, *F*′(τ, *rl*) is simply the footrule distance between all elements of τ and the corresponding elements in *rl*.
  $$\begin{array}{l} F^{\prime }\left(\tau ,rl\right)=\overset{k}{\underset{i=1}{\sum }}|\tau \left(i\right)-rl\left(i\right)| \end{array}$$
  Fagin et al. (2003) discuss another variant, *F*^(*l*)^, the footrule distance with location parameter *l*, where they set *l* = *k*+1. This is not applicable in our scenario, since we have a fixed domain and have already applied a penalty for each element τ.
- **Modified Kendall's Tau Distance**
  For a modified Kendall's Tau Distance, we again count the number of discordant pairs between τ and *rl*. This situation is similar to the modified footrule distance, as only the penalties from the elements of τ are applied.
  $$\begin{array}{l} T^{\prime }\left(\tau ,rl\right)=\underset{i,j\in P}{\sum }{\bar{T}}_{i,j}\left(\tau ,rl\right), \end{array}$$
  where *P* = {{*i, j*}|*i*≠*j* and*i, j*∈*D*}, and ${\bar{T}}_{i,j}\left(\tau ,rl\right)=1$ if *i* and *j* are in the opposite order, and 0 otherwise.

Table 2 at the end of the following section exemplifies how we use these derived rank agreement metrics adapted to our scenario to compare top-*k* lists with complete permutations in Section 6.2.

**Table 2:** Three expert opinions on the city of Munich are contrasted with the WP-jaccard ranked lists. The ranking of Expert 1 is closer to the ranked list than the two others.

	**WP-jaccard**	**Expert 1**	**Expert 2**	**Expert 3**
1	Vienna	Salzburg	Vienna	Frankfurt
2	Dusseldorf	Vienna	Milan	Brussels
3	Leipzig	Cologne	Dusseldorf	Heidelberg
4	Berlin	Graz	Paris	Budapest
5	Frankfurt	Milan	Boston	Hamburg
6	Heidelberg	Edinburgh	Luxembourg	Barcelona
7	Cologne	Dusseldorf	Berlin	Vienna
8	Nuremberg	Hamburg	Cologne	Prague
9	Salzburg	Amsterdam	Vancouver	Berlin
10	Copenhagen	Brussels	Dubai	Rome
	*F*′(τ, *m*) $\bar{x}=233.67$	146	292	263
	*T*′(τ, *m*) $\bar{x}=16.33$	14	15	20

## 5. Eliciting a Desired Concept Through Expert Opinions

Now, we want to find out which data source is best suited for the domain of destination recommendation. To do so, we have developed a web-based expert study to capture a very specific concept we are interested in: “*similar experience when visiting cities as a tourist.”* To make this latent concept explicit, we asked experts from the travel and tourism domain to give their opinion on this matter, by selecting the most similar destinations to a given city.

In a pilot study, we realized that even for experts, the task to rank the *k* most similar destinations from a list of 140 cities in world be challenging. Therefore, to obtain a sufficient number of characterizations per city, we restricted the characterization of our expert study to 50 prominent cities. Naturally, we would have preferred to perform a characterization of all cities in the data set, but given that the experts' time was limited, we focused on the 50 cities, which we expected our experts to be most familiar with.

### 5.1. Expert Survey Instrument

We now describe the user interface of the online survey application and elaborate on the design choices that influenced the system.

#### 5.1.1. Landing Page

The experts were contacted via email and, when they followed the link, they were presented with a landing page, which contained general instructions and the contact data of the authors. They were allowed to choose either from the 50 cities to be surveyed, or in the case of local experts a predefined city they should characterize.

#### 5.1.2. City Similarity Ranking Task

**Figure 3:**
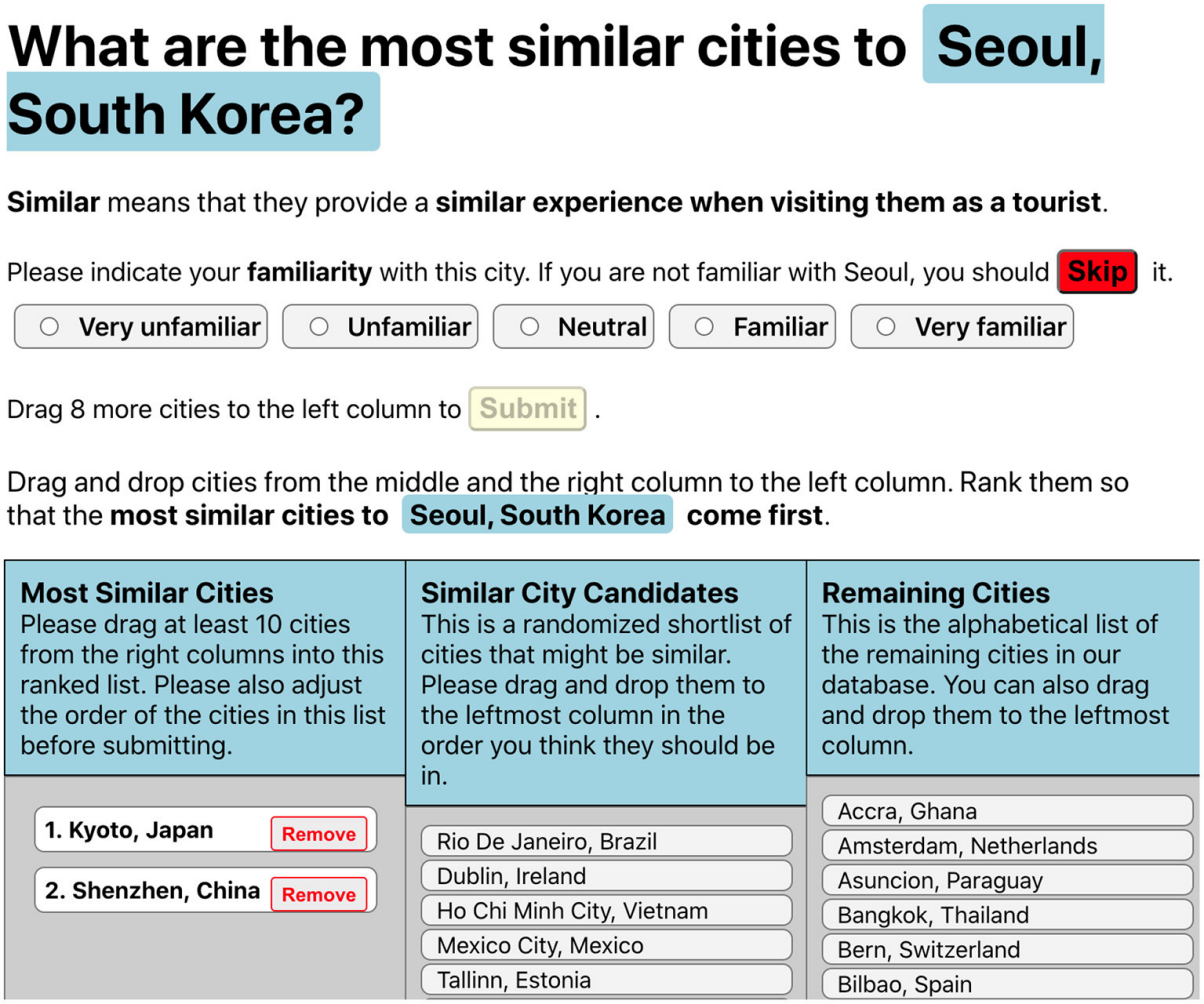
User interface of the expert survey.

After selecting the city to be characterized, the experts were presented with their task, as shown in Figure 3. First, they were asked to provide their familiarity with the given city on a five-point named Likert Scale. Then the concrete task followed, which was to be completed by ranking the cities using three columns. The left “Most Similar Cities” column was initially empty. The middle column contained a precomputed candidate list of 30 cities, that were the most similar to the base city according to the aggregation of all methods. The decision to introduce this column – as opposed to a two-column solution – was not taken lightly. It was necessary, though, since going through an unordered list of 139 items is not practical for human experts, as it would have taken a long time depleting their concentration. For this reason, we added this shortlist in a randomized order to ease the task for the experts without introducing bias in favor of a specific data source. We chose 30 as length of this shortlist, since this is three times longer than the minimum of 10 cities that needed to be dragged to the left result column. These precautions prevent biasing the results toward a specific data model. Finally, the right column contained all remaining 109 cities in alphabetical order, to provide the experts the possibility to incorporate them into their ranking.

When the experts finished dragging at least 10 cities to the left column and indicated their familiarity with the base city, a prominent “Submit” button became available. The minimum number of 10 cities was chosen to give the partial rank agreement methods sufficient information to compute meaningful results and to limit the time it takes for the experts to complete a city ranking. It also corresponds to the reality of information retrieval or recommender systems, where only few highly relevant items are of importance. When clicking the button, the results were not yet finally submitted; instead, a modal pop-up window appeared, where the users were asked to adjust the ranking of their current shortlist: “*Please adjust the order of the cities in this list before submitting.”* We decided to introduce this additional step, because when we observed test subjects in our pilot runs, it became apparent that some users simply dragged the cities into the left column without taking much care of the internal ordering of the left column. By explicitly reminding the user to revise this result, we aimed to improve the ranking, as otherwise the left column might have had set semantics instead of a ranked list semantics.

### 5.2. Sampling of Tourism Experts

To obtain a high-quality ranking data set, we reached out to experts having relevant experience with global tourism in three ways: first, we distributed leaflets to tourism experts and researchers at the ENTER eTourism Conference held in January 2020 in Guildford, United Kingdom. Second, we directly contacted representatives and researchers on the tourism boards of the 50 cities and their respective regions. Our reasoning was that these experts in the local tourism boards know best whom they compete with, and we hope this helped to establish higher diversity of where the participants of our study originated from. This group did receive a special link to the survey, which forced them to first complete the ranking of their local city, before having the chance to rank other cities as well. Finally, we also shared the user study with the TRINET Tourism Research Information Network^11^ mailing list of *accredited* members of the international tourism research and education community.

We are confident that this rigorous sampling method ensured that both the quality and the quantity of the responses are very high, despite being a web-based study conducted during the Covid-19 Pandemic.

### 5.3. Data Preparation and Cleaning

In total, we received 164 destination rankings from the survey. Since it was a web study, we took the following precautions to protect the data quality against potential low-effort submissions: We excluded responses that were completed in shorter time than 1 min and all those who did not adjust the internal ordering within the results column at all. Furthermore, we removed responses where the experts indicated their familiarity with the city on the “Very unfamiliar” or “Unfamiliar” levels. Looking at the number of completed rankings by destination, we have 28 cities with at least two submissions. Excluding the rankings of cities that were only ranked once, the results in Section 6.2 are based on the final 88 rankings of 28 cities coming from 37 different IP addresses. The characterizations done by the experts are available in the supplementary material.

The median time the experts needed to rank one city was 3m 14s and the number of re-rankings in the left results column had a median value of 4. Most submissions (74) comprised the minimum number of 10 most similar cities; eleven characterized 11–12, and the remaining three rankings were of length 13–14. The top five most characterized cities were London, UK; New York City, NY, USA; Miami, FL, USA; Barcelona, Spain; and Nice, France.

### 5.4. Example

To concertize the approach, we show the experts' rankings for the city of Munich in Table 2. The first column shows the first 10 cities of the Wikipedia-jaccard list. To obtain the score of a data model with respect to the expert's opinion, we compute the two modified rank agreement metrics between the ranked list and the experts' partial rankings. The overall score is the mean value of the rank agreement metric of all experts and all cities. The lower part of the table shows individual values and the aggregation: In this example, the opinion of Expert 1 is quite close to the ranked list according to both metrics. According to the modified Kendall's Tau, Expert 2's ranking is closer than Expert 3, however the modified Footrule distance is lower for Expert 3. This is due to the potentially exotic choices of Expert 2 to include Dubai (rank 48 in the ranked list), Vancouver (rank 54), and Boston (rank 84), which are heavily penalized in Footrule distance.

The final score of a data model according to one of the metrics is computed by the mean value of all expert rankings over all cities.

### 5.5. Expert Ranking Behavior

To provide some insights into the expert opinions, we first tabulate the number of cities that came from the 30 destination shortlist against the cities that were in the right column of Figure 3. Overall, the expert rankings comprised 80% of cities from the shortlist, whereas they still included 20% from the arguably more arduous longer list of 109 alphabetically sorted items. We see this as a confirmation that the recruited experts were serious about their task and did not only follow the ranking provided by the shortlist. Nevertheless, the shortlist might still have influenced the reviewers in a way that we cannot quantify using this study design.

In the right column of Table 3, we quantify the level of agreement among the experts. Since this ranking task is different from traditional rating data, where the agreement could be quantified using metrics such as Fleiss' kappa (Fleiss, 1971), we use a set-theoretic measure to quantify the agreement of the experts for each city. We compute the agreement as the pairwise size of the intersection over the union of two annotators. The reported number is the mean value over all pairs to make results of cities with a different number of annotators comparable. The agreement ranges between 11% in the case of Brussels, Mumbai, and Osaka, while it reaches up to 54% in the case of San Diego. On average, the experts' lists had an overlap of about 25%, which we consider as quite good, given that they chose at most 14 out of 139 other cities. Agreeing on about one-fourth of the most similar destinations both shows that there is clear common ground among the experts, but also that an intangible concept such as the touristic experience cannot be determined in a purely objective way. Finally, it should be noted that our proposed rank agreement metrics deal well with potentially diverging opinions about a concept.

**Table 3:** Expert annotators behavior: amount of cities selected from the shortlist vs. the full alphabetical list and percentages of the same cities selected.

**City name**	**Alphabetical list %**	**Shortlist %**	**Expert agreement %**
Amsterdam	20.00	80.00	17.65
Bangkok	30.43	69.57	27.78
Barcelona	40.00	60.00	12.57
Berlin	0.00	100.00	36.51
Brussels	55.00	45.00	11.11
Chicago	30.00	70.00	17.65
Copenhagen	22.73	77.27	15.79
Hamburg	23.33	76.67	27.78
Hong Kong	0.00	100.00	40.00
London	8.06	91.94	21.92
Madrid	18.00	82.00	29.50
Miami	48.00	52.00	26.59
Moscow	10.00	90.00	25.00
Mumbai	20.00	80.00	11.11
Munich	23.33	76.67	17.92
New York City	13.33	86.67	24.39
Nice	28.85	71.15	14.41
Osaka	10.00	90.00	11.11
Oslo	0.00	100.00	37.50
Paris	9.38	90.62	31.02
Rome	20.00	80.00	21.69
Saint Petersburg	43.33	56.67	17.92
San Diego	35.00	65.00	53.85
Seville	3.12	96.88	46.98
Singapore	11.63	88.37	30.72
Stockholm	12.50	87.50	32.83
Vancouver	10.00	90.00	26.32
Vienna	18.92	81.08	37.16
Overall	20.18	79.82	25.88

## 6. Results

We evaluate our work in three ways: first, an exploratory approach using pairwise comparisons of the ranked lists to capture commonalities between them. Second, the comparison of each individual data model against the top-k lists that encode the expert-elicited concept, and, finally, the results of black-box optimization of selected data sources against the expert-elicited concept.

### 6.1. Assessing the Similarity of Data Sources

**Figure 4:**
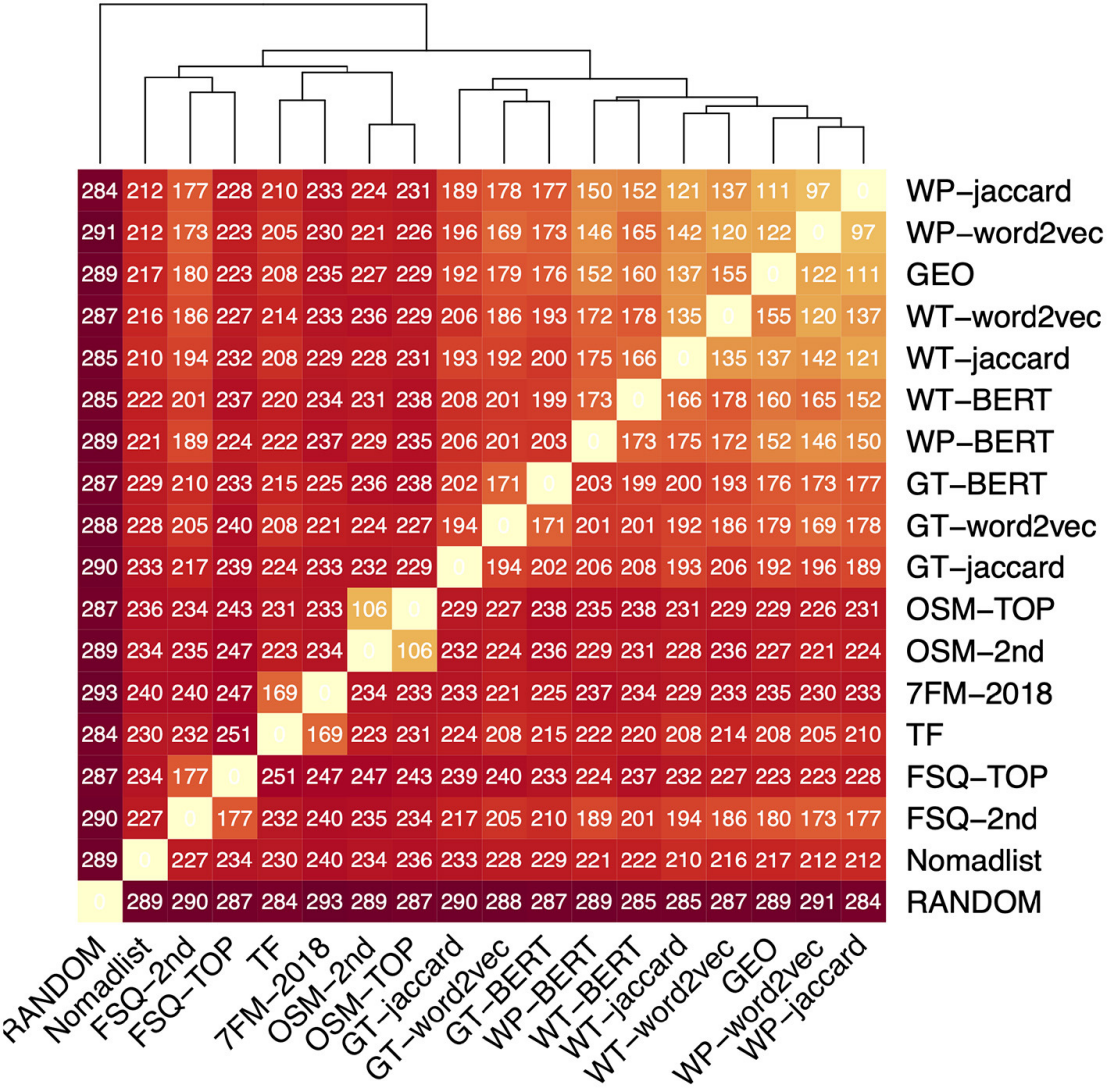
Crosswise-analysis of all data models using Kendall's Tau method. The entries are sorted using hierarchical clustering; the dendrogram reveals families of data sources. The colors are scaled according to Kendall's Tau with the bright yellow corresponding to 0 (the diagonal) and dark red representing no correlation (Random).

To reveal correlations, we compare our data sources for each city against each other using the rank agreement metrics for full permutations. In this particular case, we chose Kendall's Tau, but we could have likewise chosen Spearman's Footrule distance, which gives a similar picture. The heatmap in Figure 4 visualizes the mean pairwise distances among all ranked lists derived from the data models. The sort order was adjusted using hierarchical clustering using the Euclidean Distance, which is also the basis of the dendrogram on the top. The values in the cells are the Kendall's Tau distance, rounded to integers.

We now describe the resulting clusters. RANDOM is clearly separated from all other data models, since it has no correlation to any of them. The first group is the family of textual data sources together with GEO. The respective ranked lists (Jaccard, Word2vec, and BERT) are based on the same data source and closest to each other. Within this family, one can also see that those based on Google Travel are a bit further away from the remaining ones. We attribute the very close grouping of GEO with the ones from Wikipedia and Wikitravel to the amount of geographic information that is encoded within the articles describing the cities. Nomadlist seems to be unrelated to any other data source in particular, but unlike RANDOM still has a low correlation to all other data models. The remaining three clusters are the ones of Foursquare, OSM, and the ranked lists based on the Webologen data (TF). The high agreement between TF and 7FM-2018 is interesting, because it shows that the tourism facts are still manifested in the Seven Factor Model of the destinations. This analysis is a very compact representation of the similar concepts behind the respective data sources and their instantiation. Thus, we want to outline some further observations:

It seems that the choice of document similarity, i.e., Jaccard distance vs. cosine similarity based on word vectors, is more important in the Google Travel documents than in the Wikitravel or Wikipedia, which can be attributed to the topic, but also to the length of documents. Google Travel descriptions are around 400 words compared to 7,000 in the case of Wikitravel and 8,200 in Wikipedia.

When we compare the similarities between the top-level aggregation of OSM and Foursquare to their second-level variants, the distances are quite small between the two OSM aggregations, however, relatively large between the Foursquare aggregations. Revisiting the data, this can be explained with the very huge branching factor of the Foursquare's category tree, where the four top-level features are expanded into 337 second-level features. In the case of OSM, the four top-level categories are only expanded to 14 second-level features. This makes the Foursquare data models more dissimilar to each other than the ones from OSM.

This analysis was interesting to get a broad overview of the data sources and their commonalities. The hierarchical clustering grouped the data models in well-comprehensible families; however, the pairwise comparisons also revealed that some models that one could have expected to be quite similar, such as the top-level and second-level aggregation of Foursquare, are indeed not that similar. The benefit of this analysis is that an analyst can quickly recognize whether data models capture similar concepts to make a decision if they can be interchanged in case of them being highly correlated.

### 6.2. Comparison With the Touristic Experience

Finally, we get to answer which characterization method would be most suited to use within a content-based information retrieval system such as a destination recommender. Having elicited the concept of “*similar experience when visiting cities as a tourist”* with the expert study, we can now compare the partial rankings of the experts with our characterization methods. We use the two proposed methods from Section 4 that compare a full permutation with a top-*k* list. Furthermore, we also tabulate the Mean Reciprocal Rank (MRR), and Precision to comparison baselines. Precision@1 was very near to 0 for all characterization methods. Note that the MRR and Precision do not capture the internal rankings provided by the experts. To compute them, we treated the rankings provided by the experts as a set and aggregated the metrics over all cities included in the lists, treating each element as an individual query.

Note that the expert study is only one way to determine such a latent concept. In other domains, there are potentially different ways to elicit a baseline, but we argue that it is commonplace that a latent concept is only partially observable with respect to the set of rated items and the list of most similar items per item.

Generally, the results in Table 4 confirm the picture that was already painted in Figure 4: the versions that have shown to be similar there also rank similarly in the comparison to the expert ranking. The textual data models derived from Wikipedia, Wikitravel and Google Travel, as well as the geographic location performed best, followed by the 2nd-level aggregation of Foursquare, and the factual ones. OSM and the Foursquare top-level categories conclude the ranking with the random model unsurprisingly performing worst.

**Table 4:** Ranking of the different data sources using the modified rank agreement methods for top-*k* lists as well as MRR and Precision.

**Spearman's FR top-** ***k***		**Kendall's Tau top-** ***k***		**Mean Reciprocal Rank**		**Precision@5**		**Precision@10**	
WP-jaccard	297.011	GEO	18.284	WP-jaccard	0.101	WP-word2vec	0.186	WP-jaccard	0.304
GEO	304.091	WP-jaccard	18.750	WP-word2vec	0.101	WT-word2vec	0.182	GEO	0.302
WP-word2vec	318.080	WP-word2vec	19.068	WT-word2vec	0.100	WP-jaccard	0.178	WT-jaccard	0.297
WT-word2vec	322.489	WT-jaccard	19.227	FSQ-2nd	0.094	GEO	0.162	WT-word2vec	0.297
WT-jaccard	330.057	WP-BERT	19.568	WP-BERT	0.093	WT-jaccard	0.161	WP-word2vec	0.291
FSQ-2nd	330.420	GT-word2vec	19.852	GEO	0.093	FSQ-2nd	0.154	WP-BERT	0.279
WP-BERT	343.307	WT-word2vec	20.011	WT-jaccard	0.093	WP-BERT	0.147	FSQ-2nd	0.264
GT-word2vec	346.955	FSQ-2nd	20.625	GT-word2vec	0.088	GT-word2vec	0.139	GT-word2vec	0.263
TF	395.841	WT-BERT	20.818	WT-BERT	0.081	WT-BERT	0.139	WT-BERT	0.243
GT-BERT	396.375	TF	21.409	TF	0.075	TF	0.113	TF	0.231
WT-BERT	402.159	GT-BERT	21.477	GT-BERT	0.074	GT-BERT	0.103	GT-BERT	0.202
GT-jaccard	408.943	GT-jaccard	21.864	GT-jaccard	0.067	OSM-2nd	0.099	OSM-2nd	0.195
7FM-2018	457.909	OSM-2nd	22.375	7FM-2018	0.065	Nomadlist	0.092	GT-jaccard	0.187
Nomadlist	461.830	Nomadlist	22.420	OSM-2nd	0.065	OSM-TOP	0.090	7FM-2018	0.187
FSQ-TOP	506.500	FSQ-TOP	22.864	Nomadlist	0.063	GT-jaccard	0.087	OSM-TOP	0.180
OSM-TOP	516.114	7FM-2018	22.966	OSM-TOP	0.063	7FM-2018	0.086	Nomadlist	0.169
OSM-2nd	521.273	OSM-TOP	23.045	FSQ-TOP	0.054	FSQ-TOP	0.060	FSQ-TOP	0.122
RANDOM	649.398	RANDOM	23.341	RANDOM	0.039	RANDOM	0.033	RANDOM	0.073

The general stability of the ranking among the rank agreement metrics is high. This should not come as a surprise, since the metrics do capture the same concept; thus, we can confirm the findings of Fagin et al. (2003) that distance measures within the same equivalence class behave similarly. The absolute values of the data sources and the random baseline are quite close in some metrics, which we attribute to the small signal-to-noise ratio in the data: the rankings have only been computed on the basis of 10 – 14 items out of 140. Comparing the results to the MRR and Precision baselines, the overall trends are also similar. We again attribute this to the low signal-to-noise ratio in the evaluation of top-k lists, however, one can already see that, for example, the geographic distance becomes less successful when the ordering of the experts' lists is not taken into account.

The fact that the most successful data models according to the rank agreement with the expert study stem from freely available textual descriptions of the destinations, as well as the geographic location, is an interesting finding. The good result of the geographic location can be explained using the intuition that nearby destinations are often within a similar culture and climate and, thus, also have a similar experience when visiting them according to our expert rankers. The articles in the Wikipedia and Wikitravel also do a good job of emulating the expert-elicited concept. Many travelers already use such sources to inform themselves about potential destinations and we attribute the consistently higher ranking of the Wikipedia over Wikitravel to the different target audiences. As a travel guide, Wikitravel is more oriented toward travelers already at the destination seeking practical travel information such as restaurant suggestions, while the Wikipedia offers a more comprehensive overview of the culture, history, and attractions of a city.

We now can also see that the differences between the two document similarities, cosine similarity based on word vectors and the Jaccard Distance, do matter with respect to the baseline. For the shorter Google Travel documents, the word embeddings outperformed their counterpart, whereas the Jaccard Distance was slightly better for the longer Wikipedia and Wikitravel texts. We attribute the lesser performance of the BERT transformer encoder architecture due to the fact that the touristic information is mostly encoded within the terms, thus, using full contextual embeddings does not benefit the performance of the characterization.

When looking at the expressiveness of the data sources, we see no connection between the amount of information that is explicitly encoded within the features of a data model and its performance. This suggests that more information is not needed to build a successful data model, but features that are of high quality with respect to the target concept. The highly successful geographic distance only consists of two floating numbers [−180;180], but of course, implicitly encodes much relevant information for travelers such as the culture and climate of a city. As we will see in the next section, this analysis can be used to improve the performance of some data models by dropping features that are not useful toward the target domain.

Why were the factual and venue-based destination characterization methods, of which some are already employed in destination recommender systems (Sertkan et al., 2019; Myftija and Dietz, 2020) outperformed? The reason lies within the very specific concept that we elicited using the expert survey. The factual and venue-based data models could not have been optimized toward the concept of “touristic experience” based on the insights from the survey, since when constructing them, the respective authors had no instantiation of the concept available or potentially decided to optimize toward a different concept. For example, the Nomad List characterization is aimed at digital nomads instead of tourists. Thus, it would have been be surprising if it was in a front runner position, as digital nomads have different information needs than a typical tourist does. For the same reason, the OSM performed quite poorly. Instead of the touristic experience, they simply captured the similarity of the distribution of the different map entities. On the contrary, the textual data sources are there to learn about the characteristics of a city, so it is not surprising that they encode the most useful information for travelers. This means that the proposed methods are able to discriminate between similar and somewhat orthogonal concepts and do so by quantifying the distance. Since some features of Nomad List, OSM, and Foursquare were not aimed at encoding the same concept that we have elicited in the expert study, it is just natural that these data sources perform underwhelmingly in our initial comparison. Our methods reveal the degree to which the expert-elicited concept is not (well) encoded within the features, but since the characterizations are somewhat related to traveling, they are not orthogonal.

To summarize, the proposed rank agreement metrics for top-*k* lists have been successfully employed in determining the quality of the data sources with respect to the expert-elicited concept. They produce comparable rankings as established information retrieval metrics, such as MRR and Precision. The advantage is that rank agreement metrics operate on ranked lists instead on sets, making them conceptually more fitting than MRR, and Precision or similar metrics.

### 6.3. Optimization of Data Models

With this tooling established, there is now potential to refine existing data models based on tourism facts and the venue distributions by learning the importance of the respective features or even constructing a composite data model with features from different data sources. By assigning different weights to the features based on their importance in computing similarity metrics, rich models with several features can be fine-tuned toward the expert-elicited concept. This is useful, since standard similarity metrics in content-based recommendation, such as the Euclidean Distance give same weight to all features. In practice, however, not all features equally contribute to the expert-elicited concept of the touristic experience. By decreasing the weights of less-relevant features, the similarity metric can be improved to emulate the expert concept even better.

Given the combinatorial explosion of the search space for weights, we have used black-box learning, namely Simulated Annealing (Kirkpatrick et al., 1983) for tuning the weights [0,1] of the data sources with explicit features. The proprietary TF and 7FM-2018 sources were only provided to us as rankings, thus, we could not optimize those.

The optimization tabulated in Table 5 works better with more features, as can be seen with Nomadlist, FSQ-2nd, and OSM-2nd. We attribute the small relative changes in FSQ-TOP and OSM-TOP to the fact that they capture slightly orthogonal concepts to the expert-elicited baseline and due to their smaller number of features, they are harder to optimize toward this concept. However, as discussed before, this domain has a high signal-to-noise ratio, making these small relative improvements relevant in the overall comparison. Concretely, the optimized version of FSQ-2nd would be the third most competitive data model in Table 4.

**Table 5:** Optimization toward the Expert Opinion using Spearman's Footrule top-*k*.

**Model**	**Unoptimized**	**Optimized**	**Improvement**
Nomadlist	461.83	426.27	7.70%
FSQ-TOP	506.50	503.33	0.63%
FSQ-2nd	330.42	312.47	5.43%
OSM-TOP	516.11	508.38	1.50%
OSM-2nd	521.27	490.33	5.94%

What is more, even with minor contributions to the overall performance, the learned weights for the features gives further insight into their importance. Features with a very low weight could be dropped, while the feature selection of a potential combined data model of several data sources should be guided by the learned weights.

To exemplify the insights from this analysis, we discuss the learned weights of the Nomadslist data: The features “cost,” “life quality,” “air quality,” and “happiness” got relatively high values ranging from 0.58 to 0.75, while the other features, “nomad score,” “fun,” “healthcare,” and “nightlife” were reduced to low weights ranging between 0.2 and 0.35. Such low values indicating that they are not in line with the elicited concept of the expert study. In FSQ-TOP, which is employed in the CityRec system (Dietz et al., 2019), “food” gets a very low weight, which is an indication that it could be dropped from the recommendation algorithm.

The results of this in-depth analysis of the weights are certainly quite specialized with respect to the target concept and the intricacies of the respective data sources. For this reason, we do not further elaborate on the other optimized models but refer the reader to the full results of this optimization tabulated in the reproducibility material. The method, however, is again generalizable for any domain, where the data source's features are known and a baseline exists in the form of ranked lists.

## 7. Conclusions

We presented a comprehensive overview of data-driven methods to characterize cities at scale using online data. Motivated by the question of model choice in destination recommender systems, we proposed methods to make such data models of destinations comparable against each other as well as against a – potentially latent – concept, that the recommender system should emulate when computing content-based recommendations. To derive this concept, we conducted an expert study that provided us with partial rankings and provided us opportunity to further optimize the data models that are based on explicit features. The decision of eliciting this baseline using experts instead of large-scale crowd-sourcing was done due to the difficulty of the task. Since this is the first study analyzing latent concepts encoded in features of content-based recommender systems, we decided to elicit a high-quality data set with less noise, than having a large-scale data set that is less to be trusted.

In a first step, we were able to unveil commonalities of data sources, through which it became apparent that, for example, articles about destinations on Wikipedia and Wikitravel encode much geographic information. The second contribution are methods to compare top-*k* lists with permutations in our specific scenario. We used these to show that, according to the expert opinion, the touristic experience was best approximated using the textual similarities from Wikipedia, Wikitravel, and the geographic location. This means that when simply retrieving the most similar destinations according to the touristic experience, one can choose one of the top-ranked entries from Table 4. Finally, we were able to show that it is possible to optimize the distance metric of a content-based recommender system toward a desired concept.

From a recommender systems research perspective, the results show that existing destination recommender systems do not necessarily use data models that capture the concept of similar touristic experience very well. This might be intentional, if the system's purpose is to capture a different concept, or possibly due to the previous lack of a concrete instantiation of the concept. A limitation of the top-ranked textual or geographic characterizations is that they do not come with specific features the user can interact with. This is a drawback, since it means that they cannot directly place the user's preferences and the items in a common vector space to perform content-based recommendation as frequently done in travel recommender systems (Burke and Ramezani, 2011). Furthermore, common recommendation techniques such as critiquing (Chen and Pu, 2012), i.e., giving a system feedback about the features of a suggested item, are only possible if the items are characterized with a fixed number of features.

Our work has provided the community with adequate tools to optimize feature-based data models toward a desired concept such as the similar touristic experience. The methodological contribution, is, however, not limited to recommender systems in the tourism domain, but can be applied in other domains similarly as the proposed metrics operate on ranked lists. Latent similarity concepts are prevalent in many domains such as music (Yoshii et al., 2006) or leisure activities (Brítez, 2019); generally anywhere, where the accuracy of the information retrieval system depends on the embeddings of items in a search space.

A logical continuation of this work would be to investigate the potential to construct better, potentially combined data models. This research can help to improve all kinds of data-driven characterizations of travel destinations as it provides direct feedback about the data quality with respect to the touristic experience. While this time we used an expert study, we also plan to apply these methods in other domains in a large-scale crowd-sourcing setting. Finally, it would be worthwhile to perform an analysis of the effect of improved data model quality with respect to further evaluation metrics such as accuracy.

## Data Availability Statement

The datasets presented in this study can be found in online repositories. The names of the repository/repositories and accession number(s) can be found below: https://github.com/LinusDietz/destination-characterization-replication.

## Ethics Statement

Ethical review and approval was not required for the study on human participants in accordance with the local legislation and institutional requirements. The patients/participants provided their written informed consent to participate in this study.

## Author Contributions

LD has compiled the data, developed the metrics, conducted the analyses, and was the main author of the manuscript. MS contributed various data sources and co-wrote the manuscript. SM contributed two data models and was the main developer of the expert survey instrument. ST contributed two data models. JN recruited domain experts and together with WW supervised the project. All authors read and approved the final manuscript.

## Conflict of Interest

The authors declare that the research was conducted in the absence of any commercial or financial relationships that could be construed as a potential conflict of interest.

## Publisher's Note

All claims expressed in this article are solely those of the authors and do not necessarily represent those of their affiliated organizations, or those of the publisher, the editors and the reviewers. Any product that may be evaluated in this article, or claim that may be made by its manufacturer, is not guaranteed or endorsed by the publisher.

## Footnotes

^1^ https://www.google.com/streetview/

^2^ https://developer.foursquare.com/docs/api/venues/search

^3^ https://developer.foursquare.com/docs/resources/categories

^4^ https://wiki.openstreetmap.org/wiki/Map_Features

^5^ https://en.wikipedia.org

^6^ https://wikitravel.org

^7^ https://www.google.com/travel

^8^ https://spacy.io

^9^ https://commoncrawl.org

^10^ https://nomadlist.com

^11^ https://tim.hawaii.edu/about-values-vision-mission-accreditation/trinet/

## Abbreviations

LBSN: location-based social network
OSM: OpenStreetMap.
